# An HDAC3-PROX1 corepressor module acts on HNF4α to control hepatic triglycerides

**DOI:** 10.1038/s41467-017-00772-5

**Published:** 2017-09-15

**Authors:** Sean M. Armour, Jarrett R. Remsberg, Manashree Damle, Simone Sidoli, Wesley Y. Ho, Zhenghui Li, Benjamin A. Garcia, Mitchell A. Lazar

**Affiliations:** 1 Institute for Diabetes, Obesity, and Metabolism, Perelman School of Medicine at the University of Pennsylvania, 3400 Civic Center Boulevard, SCTR 12-102, Philadelphia, PA 19104 USA; 2 Divison of Endocrinology, Diabetes, and Metabolism, Department of Medicine, Perelman School of Medicine at the University of Pennsylvania, 3400 Civic Center Boulevard, SCTR 12-102, Philadelphia, PA 19104 USA; 3 Department of Biochemistry and Biophysics, Perelman School of Medicine at the University of Pennsylvania, 3400 Civic Center Boulevard, SCTR 12-102, Philadelphia, PA 19104 USA

## Abstract

The histone deacetylase HDAC3 is a critical mediator of hepatic lipid metabolism, and liver-specific deletion of HDAC3 leads to fatty liver. To elucidate the underlying mechanism, here we report a method of cross-linking followed by mass spectrometry to define a high-confidence HDAC3 interactome in vivo that includes the canonical NCoR–HDAC3 complex as well as Prospero-related homeobox 1 protein (PROX1). HDAC3 and PROX1 co-localize extensively on the mouse liver genome, and are co-recruited by hepatocyte nuclear factor 4α (HNF4α). The HDAC3–PROX1 module controls the expression of a gene program regulating lipid homeostasis, and hepatic-specific ablation of either component increases triglyceride content in liver. These findings underscore the importance of specific combinations of transcription factors and coregulators in the fine tuning of organismal metabolism.

## Abstract (web summary)

HDAC3 is a critical mediator of hepatic lipid metabolism and its loss leads to fatty liver. Here, the authors characterize the liver HDAC3 interactome in vivo, provide evidence that HDAC3 interacts with PROX1, and show that HDAC3 and PROX1 control expression of genes regulating lipid homeostasis.

## Introduction

Hepatic lipid homeostasis is critical for the maintenance of normal liver physiology and organismal metabolism. Lipid composition and accumulation in the liver is controlled by a complex network of interconnected metabolic pathways such as lipid synthesis, lipolysis, β-oxidation, secretion, and storage, and the dysregulation of even one of these pathways can lead to lipid accumulation in liver or hepatic steatosis^1, 2^. Non-alcoholic fatty liver disease (NAFLD), defined by excess fat in the liver, is of growing clinical relevance in industrialized countries and is a major risk factor for the development of non-alcoholic steatohepatitis, liver fibrosis, and cirrhosis^3^. In addition, NAFLD has been linked to cardiovascular disease, metabolic syndrome, insulin resistance, and hepatocellular carcinoma^2^. Thus, the mechanisms governing liver lipid homeostasis are of broad importance to understanding the development of NAFLD and to identify targets for therapeutic intervention.

These pathways of lipid homeostasis are regulated in liver by a host of transcription factors including the nuclear receptors HNF4α^4, 5^, Rev-erbα^6^, LXRs^7^, PPARs^8^, and the E-box binding proteins SREBP and ChREBP^9^ among others. In addition to these sequence-specific DNA-binding factors, numerous cofactors and coregulators have been shown to influence the expression of genes controlling lipid metabolism^10^. Previous work has demonstrated histone deacetylase 3 (HDAC3) to be an important epigenomic coregulator in liver^6, 11^, and deletion of HDAC3 in adult liver results in remarkable hepatic steatosis^12^. However, the fundamental mechanisms of how HDAC3 controls metabolic gene transcription in liver are not completely understood.

HDAC3 is unique among the class I histone deacetylases as it requires binding to the nuclear receptor corepressor (NCOR1)^13^ or the silencing mediator for retinoic acid and thyroid hormone receptors (SMRT or NCOR2)^14^ for its enzymatic activity^15–17^. Together with transducing β-like 1X-linked and receptor 1 (TBL1X and TBL1XR1)^18^ and the G-protein suppressor 2 (GPS2)^19^, these proteins form the core of the NCoR transcriptional repressor complex^20^. The NCoR complex has been shown to be a major corepressor complex for the nuclear receptor family of transcription factors^13, 14, 20, 21^. Deletion of individual components of the NCoR complex results in an increase in liver triglycerides^12, 22, 23^, highlighting the importance of these proteins acting together as a functional complex to regulate liver metabolic gene transcription.

The vast number of transcriptionally relevant complexes highlights the important roles protein–protein interactions play in the control of gene expression. For HDAC3, there are important questions about which transcription factors recruit it to the genome, and which HDAC3-associated proteins act as downstream effectors to impact lipid gene regulation and hepatic steatosis.

Here, we describe NEAT ChIP-MS (Nuclear Extraction Affinity Tag), an improved chromatin cross-linking method followed by nano liquid chromatography-tandem mass spectrometry (nLC-MS/MS) analysis to identify in vivo interactions in liver and define a high-confidence interactome for HDAC3. We find a strong association between HDAC3 and the Prospero-related homeobox 1 protein (PROX1), which co-localize at the genome and at some sites are completely dependent on the nuclear receptor HNF4α for their co-recruitment. Interestingly, depletion of PROX1 in liver results in increased hepatic triglycerides similar to loss of HDAC3. Our results suggest an important role for an HDAC3–PROX1 corepression module in regulating the transcription of a gene program important for the maintenance of lipid homeostasis.

## Results

### In vivo screen for HDAC3 interactors

**Fig. 1:**
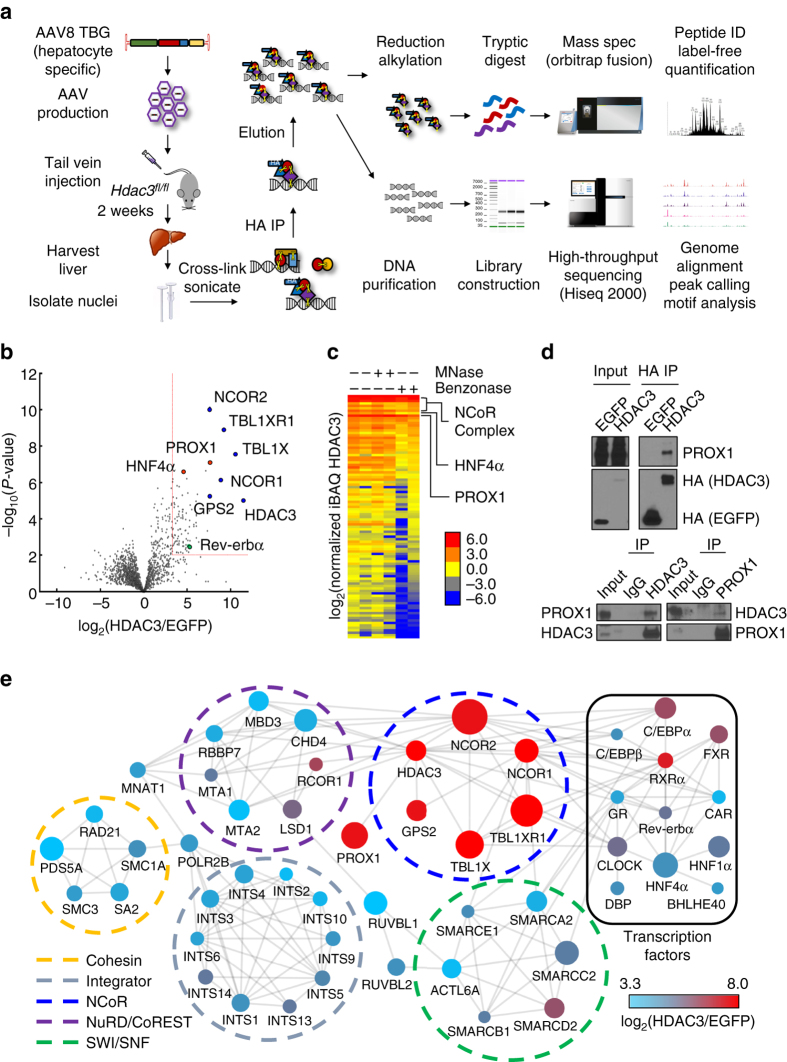
NEAT ChIP-MS reveals the HDAC3 liver nuclear interactome. **a** Schematic illustration of the NEAT ChIP-MS protocol. HA-tagged HDAC3 or EGFP was expressed with or without *Cre* in *Hdac3* ^fl/fl^ mice specifically in hepatocytes via adeno-associated virus (AAV8 TBG). After isolation of nuclei and cross-linking with formaldehyde, HDAC3 or EGFP control were captured by anti-HA immunoprecipitation. Protein complexes and associated DNA sequences were analyzed by mass spectrometry or high-throughput sequencing, respectively. **b** Volcano plot of mass spectrometry analysis of HDAC3 interacting proteins (HDAC3 *n* = 11, EGFP *n* = 13). The *x* axis indicates log_2_ ratio of normalized intensity (iBAQ) of proteins discovered in HDAC3 to EGFP control. *Red box* indicates fold-change (10-fold) and *P*-value (0.01) cutoffs for interactors. Core NCoR complex components (*blue*), selected high scoring interactors (*red*), and Rev-erbα (*green*) are indicated. **c** Heatmap of normalized intensity (iBAQ) of HDAC3 interactors from **b** in the presence or absence of micrococcal nuclease (MNase) or Benzonase nuclease. Each lane represents an independent experiment. **d** Co-IP experiments confirming interaction of HDAC3 with PROX1 from liver expressing tagged HDAC3 (*top*) or endogenous IPs (*bottom*). **e** Protein–protein interaction network analysis. *Circle color* represents enrichment over control and the *size of the circle* represents −log_10_(*P*-value). *Nodes* displayed met eightfold enrichment cutoff after Benzonase treatment and *lines* indicate validated interactions (STRING, active interaction sources include experiments and databases, minimum interaction score 0.6). Interactors are grouped by known type and *circles* indicate known functional complexes

To elucidate nuclear interactors of HDAC3 in vivo, we developed NEAT ChIP-MS, a cross-linking proteomic interaction method that allowed for the confident identification of HDAC3 interactors in adult liver (Fig. 1a). *Hdac3* ^*fl*/*fl*^ animals were tail vein-injected with either AAV virus expressing epitope-tagged HDAC3 (AAV8 TBG HDAC3-HA) in conjunction with AAV8 TBG Cre to deplete endogenous HDAC3 in hepatocytes as described^12^, or with control virus expressing epitope-tagged green fluorescent protein (AAV8 TBG HA-EGFP). Subsequent to nuclear isolation, samples were cross-linked and EGFP or HDAC3 was immunoprecipitated with anti-HA resin. Associated proteins were analyzed by nLC-MS/MS and the results of the HDAC3 and EGFP interactomes were compared to remove non-specific interactions. We applied stringent significance (*P* < 0.01) and fold enrichment (10-fold) cutoffs using label-free quantification values estimated by the intensity-based absolute quantification (iBAQ)^24^ algorithm to the resultant interactome to identify a set of high-confidence HDAC3 interacting proteins in vivo in mouse liver (Fig. 1b and Supplementary Data 1).

As expected, we observed an enrichment of all the components of the previously identified NCoR complex (sequence coverage indicated in parentheses) consisting of NCOR1/2 (11.14%/12.14%), TBL1X (46.62%), TBL1XR1 (42.52%), GPS2 (4.33%), and HDAC3 (42.85%) itself. In addition, our screen confirmed interaction of HDAC3 with a number of transcription factors known to interact with the NCoR complex, including the circadian nuclear receptor Rev-erbα^6^. One of the most significantly enriched HDAC3 interactors, but not considered a core component of the NCoR complex, was the Prospero-related homeobox 1 (PROX1). A highly conserved transcription factor in vertebrates, PROX1 was previously shown to be critical for the development of several organs including the lymphatic system^25^, lens^26^, liver^27, 28^, pancreas^28^, heart^29^, and skeletal muscle^30^. In addition, PROX1 has been implicated in regulating the functions of several nuclear receptors^31–36^ and has been shown to act as both a transcriptional repressor^31–33^ and activator^26, 37^ in different biological contexts.

To assess whether the proteins discovered in our chromatin bound complexes purified with HDAC3 directly through protein–protein interactions or indirectly through close genomic proximity, we treated our lysates with either micrococcal nuclease (MNase), capable of cutting only in nucleosome-free regions, or Benzonase nuclease, which can cleave DNA regardless of nucleosome occupancy. Although micrococcal nuclease had minimal impact, Benzonase nuclease significantly reduced the intensity of a subset of protein–protein interactions with HDAC3 (Fig. 1c and Supplementary Data 2). This suggested that these proteins are not in direct contact with HDAC3, and thus these protein–protein associations are likely facilitated by DNA. In addition, interactors associated with HDAC3 exhibiting lower enrichments tended to be more susceptible to Benzonase treatment, implying that proteins displaying greater abundance were, in general, more likely to occur through protein–protein interactions. The group of proteins maintained upon nuclease treatment included PROX1, indicating that this robust HDAC3 interactor was likely more directly bound and not dependent on DNA bridging for its interaction.

The interaction of PROX1 and HDAC3 in liver was confirmed by coimmunoprecipitation experiments using the reversible cross-linker DSP from livers expressing HA-tagged HDAC3 or endogenously from wild-type livers (Fig. 1d). PROX1 and HDAC3 interaction likely occurs in the context of the NCoR complex, as interaction between PROX1 and NCOR1 was observed in cell culture, and also requires multiple surfaces in the N terminus of PROX1 for binding (Supplementary Fig. 1a, b). We next sought to better understand the HDAC3 interactome from a more global functional perspective. Classifying the interactors and their biological links and annotating them using information from the STRING database revealed several interesting clusters of proteins associating with HDAC3 (Fig. 1e). In addition to the NCoR complex, HDAC3 was found to associate with several other transcriptionally important complexes including SWI/SNF, Integrator, Cohesin, and components of the NuRD and CoREST repressor complexes. We also observed a number of sequence-specific DNA-binding transcription factors of functional relevance to liver physiology such as C/EBPs, FXR, RXRα, and HNF4α. We also utilized cytoHUBBA, a tool to define network topology^38^, and identified members of the NCoR complex, NuRD/CoREST complex, Integrator complex, and nuclear receptors as important nodes in the liver HDAC3 interactome (Supplementary Fig. 2). These results indicate that HDAC3 likely has an important role in regulating the expression of liver gene programs through interaction with an array of transcription factors and suggest that PROX1, as a strong interactor in liver, may be of more general importance to the functionality of HDAC3.

### PROX1 and HDAC3 co-localize extensively at the genome

**Fig. 2:**
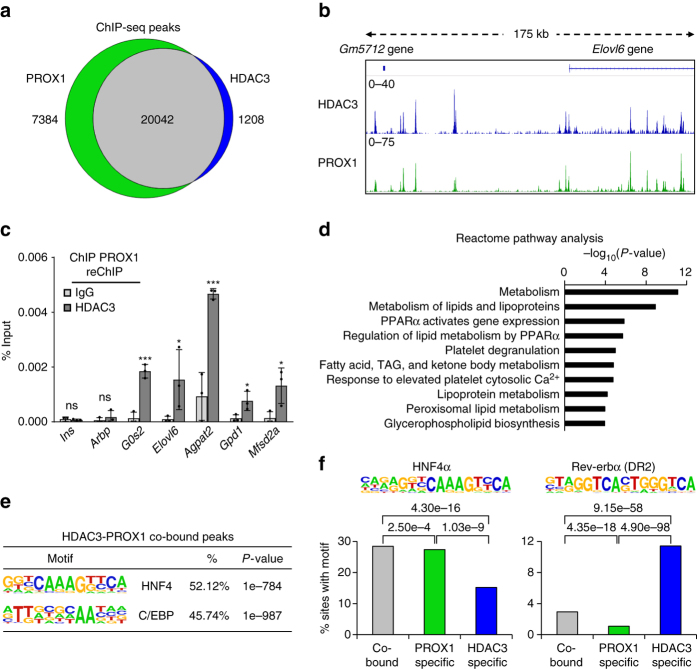
HDAC3 and PROX1 exhibit extensive co-binding and reveal a metabolic signature. **a** Venn diagram displaying overlap of peaks identified in PROX1 (2 rpm cutoff) and HDAC3 (1.5 rpm cutoff) ChIP-seq. Peaks required 50% overlap and have a minimum 1 rpm signal for the other factor. **b** Representative browser tracks of HDAC3 and PROX1 ChIP-seq. *Scale* is reads per ten million (RPTM). **c** Co-occupancy of PROX1 and HDAC3 as indicated by ChIP–reChIP (*n* = 3) from liver. *Legend* indicates reChIP antibody following primary PROX1 ChIP elution. Data are presented as mean ± s.d., one-tailed unpaired Student’s *t*-test, *P < 0.05, **P < 0.01, ***P < 0.001, *ns* not significant. **d** Reactome analysis of the nearest genes within 100 kb from the top 1000 overlapping HDAC3 and PROX1 peaks. **e** HOMER motif analysis of co-bound peaks displaying over-represented sequences. **f** HOMER motif enrichment analysis of the indicated motifs (HNF4α and Rev-erbα DR2) at overlapping and non-overlapping peaks determined in (**a**). *Numbers above brackets* indicate *P*-values, *χ* ^2^ test

To further investigate the functional interplay between PROX1 and HDAC3, we performed ChIP-seq on PROX1 and HDAC3 in mouse liver. Bioinformatic analysis of the peaks discovered in the two data sets revealed a strong overlap between the binding sites of the HDAC3 and PROX1 cistromes (Fig. 2a). Visual inspection of individual ChIP-seq tracks confirmed a striking correlation (Fig. 2b). We next performed ChIP–reChIP analysis, which demonstrated that both HDAC3 and PROX1 were co-bound to the same DNA fragments (Fig. 2c). Analysis of overlapping peaks by pathway analysis (Reactome) revealed that co-bound sites were enriched at genes involved in a variety of important metabolic pathways, including those that regulate lipid metabolism (Fig. 2d). As expected peaks bound by HDAC3 selectively showed enrichment both for liver metabolic genes and those involved in circadian rhythm, indicating that HDAC3 likely has functions with Rev-erbα independent of its interaction with PROX1 (Supplementary Fig. 3a). HOMER motif analysis of the overlapping peaks displayed a strong enrichment for the HNF4 and C/EBP motifs, two transcription factors that are known to have important roles in liver development and adult liver function (Fig. 2e). Upon closer examination of overlapping, HDAC3-only, and PROX1-only peaks, we observed that the HNF4α motif was significantly reduced at HDAC3-only peaks, whereas the Rev-erbα (DR2) motif was significantly enriched at these sites (Fig. 2f). The C/EBP motif followed a similar pattern observed for HNF4α indicating that it may also have a role in coordinating these sites but not those involved in circadian function (Supplementary Fig. 3b). These results suggest that HDAC3 is recruited to at least two subsets of sites, ones that contain only HDAC3, which are recruited by Rev-erbα, and those that have both PROX1 and HDAC3, and are recruited by HNF4α.

### HNF4α is required for recruitment of the HDAC3–PROX1 module

**Fig. 3:**
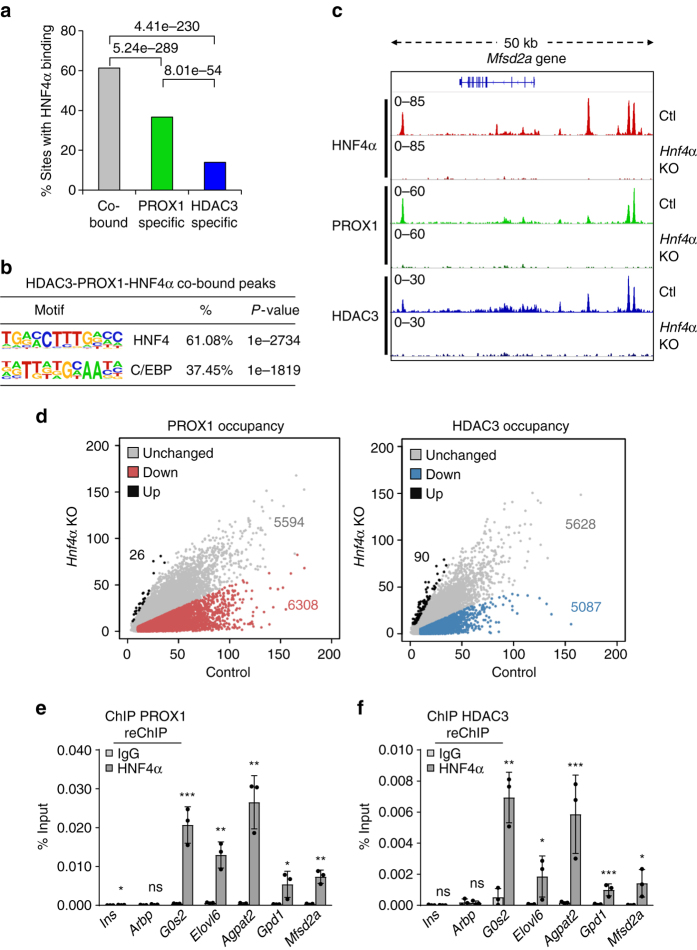
The HDAC3–PROX1 module is recruited by HNF4α in liver. **a** Comparison of HNF4α binding (>2 rpm, filtered on the HNF4α KO, 50% minimum overlap) at overlapping and non-overlapping peaks in the HDAC3 and PROX1 cistromes from Fig. 2a. *Numbers above brackets* indicate *P*-values, *χ* ^2^ test. **b** HOMER motif analysis of peaks co-bound by HDAC3, PROX1, and HNF4α displaying over-represented sequences. **c** Representative browser tracks of HNF4α, PROX1, and HDAC3 ChIP-seq in *Hnf4α* ^fl/fl^ livers infected with AAV8 TBG *Egfp* (Ctl) or *Cre* (*Hnf4α* KO). Indicated *scales* are in RPTM. **d** Scatter plots of PROX1 (*left*) and HDAC3 (*right*) ChIP-seq in control versus *Hnf4α* KO. *Red* and *blue shaded regions* indicate a twofold decrease in peak intensity upon loss of HNF4α. **e**, **f** Co-occupancy of HDAC3–PROX1 and HNF4α as indicated by ChIP–reChIP (*n* = 3) from liver. *Legend* indicates reChIP antibody following primary HDAC3 or PROX1 ChIP elution. Data are presented as mean ± s.d, one-tailed unpaired Student’s *t*-test, *P < 0.05, **P < 0.01, ***P < 0.001, *ns* not significant

Given our observation that the HNF4α motif was found prominently at co-occupied sites, we hypothesized that this important liver transcription factor may have a role in the recruitment of HDAC3 and PROX1. ChIP-seq analysis of HNF4α in control liver demonstrated HNF4α binding at >60% of HDAC3–PROX1 co-bound peaks (Fig. 3a). In stark contrast, HDAC3-only peaks displayed significantly reduced HNF4α binding (~14%). Motif analysis of the triply bound peaks showed an increased enrichment of the HNF4 motif and a mild de-enrichment of the C/EBP motif when compared to all HDAC3–PROX1 co-bound peaks (Fig. 3b). To address whether HNF4α was required for the recruitment of HDAC3–PROX1 at the co-bound sites we injected *Hnf4α* ^*fl*/*fl*^ mice with AAV8 TBG Cre to delete *Hnf4α* in hepatocytes (Supplementary Fig. 4a). ChIP-seq analysis of HNF4α in these mice revealed near complete loss of HNF4α binding at the genome (Fig. 3c and Supplementary Fig. 4b–d). Remarkably, a significant portion of both HDAC3 and PROX1 peaks were strongly reduced upon loss of HNF4α (Fig. 3d). Moreover, the HDAC3 and PROX1 sites that were affected were almost exclusively the same sites (Supplementary Fig. 5a).

Co-binding of both PROX1 and HDAC3 with HNF4α nearby several lipid-related genes were confirmed by ChIP–reChIP (Fig. 3e, f). In addition, we observed a decreased interaction of PROX1 and HDAC3 in the *Hnf4α* knockout mice by Co-IP western blot, suggesting HNF4α may be required to stabilize the HDAC3–PROX1 interaction in vivo (Supplementary Fig. 5b). We compared the binding of both HNF4α and C/EBPβ at the co-bound sites that were lost in *Hnf4α* knockout versus those that were unaffected and observed a positive relationship between the strength of HNF4α binding and the dependency of HDAC3/PROX1 on HNF4α (Supplementary Fig. 5c). C/EBP, serving as a control comparison, did not display a similar correlation. These results strongly suggest that HNF4α recruits the HDAC3–PROX1 module to a substantial subset of binding sites, whereas other factors, like C/EBP, may be more important elsewhere in the genome.

### Liver depletion of PROX1 increases hepatic triglycerides

**Fig. 4:**
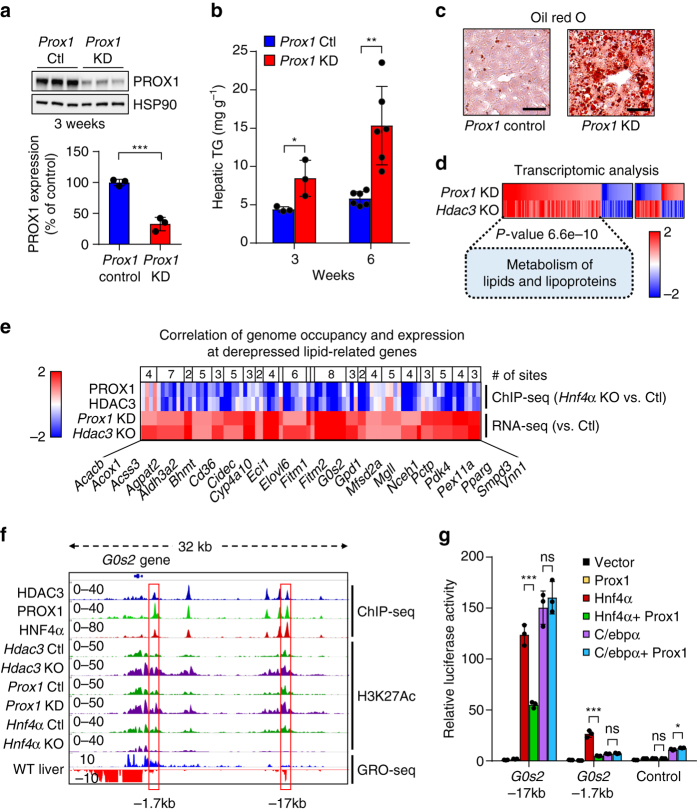
The HDAC3–PROX1 module suppresses steatosis by controlling a hepatic lipid metabolism gene program. **a** Western blot of liver samples from mice treated for 3 weeks with AAV8 TBG shLuciferase (*Prox1* Ctl) or shPROX1 (*Prox1* KD). Densitometry of the western blot shown above (*n* = 3). Data are presented as mean ± s.d. **b** Hepatic triglyceride assay of livers infected for 3 weeks (*n* = 3) or 6 weeks (*n* = 6) with indicated virus. Data are presented as mean ± s.d. **c** Oil red O staining of livers infected for 6 weeks as in (**b**). *Scale bar* is 50 µm. **d** RNA-seq analysis of *Hdac3* ^fl/fl^ mice infected for 2 weeks with AAV8 TBG *Egfp* (*n* = 2) or *Cre* (*Hdac3* KO, *n* = 3) vs. wild-type mice infected with AAV8 TBG shLuciferase (*n* = 3) or shPROX1 (*Prox1* KD, *n* = 3) for 3 weeks. Heatmap displays coregulated genes (1.7-fold change, *P* < 0.05) grouped by expression correlation (135 genes) or anti-correlation (50 genes) upon ablation of HDAC3 or PROX1. *Scale bar* represents log_2_(fold change). *Inset* shows the *P*-value and corresponding gene list for the highest ranking Reactome pathway in the co-upregulated cluster. **e** Heatmap displays co-upregulated lipid-related genes upon ablation of HDAC3 or PROX1 and the corresponding binding strength of adjacent HDAC3–PROX1 co-bound peaks (−50kb upstream of the transcription start site, TSS through +2 kb from the transcription end site, *TES*) in the HNF4α KO liver relative to control. *Scale bar* represents log_2_(fold change) for RNA-seq and fold-change for ChIP-seq. **f** Example ChIP-seq and GRO-seq browser tracks at the *G0s2* locus. *Red boxes* indicate location of putative *G0s2* enhancers. **g** Luciferase assay (*n* = 3) indicating transcriptional response to co-expression of HNF4α, PROX1 and C/EBPα at *G0s2* enhancers as identified in (**f**). Data are presented as mean ± s.d. Two-tailed unpaired Student’s *t*-test, *P < 0.05, **P < 0.01, ***P < 0.001, *ns* not significant

To determine the function of PROX1 in liver, *Prox1* was knocked down in adult hepatocytes of wild-type male mice using an AAV-based shRNA delivery vector (AAV8 TBG shProx1), which led to a ~70% depletion in PROX1 protein 3 weeks following injection (Fig. 4a). Importantly, no significant change in HDAC3 protein levels were observed upon knockdown of PROX1 (Supplementary Fig. 6a). Hepatic triglycerides (TG) were significantly elevated following 3 weeks of PROX1 knockdown, with a more striking difference in TG levels observed at 6 weeks post infection (Fig. 4b). Oil red O staining confirmed an increase in neutral lipid in livers of male mice infected with *Prox1* shRNAs (Fig. 4c). Thus, similar to HDAC3 knockout, depletion of PROX1 in liver results in increased triglyceride content. Of note, hepatic PROX1 levels were not significantly changed after 12 weeks of high fat diet (Supplementary Fig. 6b). Moreover, knockdown of *Prox1* in female livers led to a modest but non-significant increase in TG, potentially indicating a gender-specific response (Supplementary Fig. 6c).

### HDAC3 and PROX1 coregulate a lipid gene expression program

To determine if transcriptional mechanisms of coregulation by HDAC3 and PROX1 had a role in regulating this striking liver lipid phenotype, we performed RNA-seq on *Hdac3* knockouts (Supplementary Fig. 6d) and 3 week *Prox1* shRNA livers. We observed a significant overlap of genes whose expression was altered by loss of either factor, with a striking over-representation of genes whose directionality of change was correlated and a strong preference for upregulated genes, consistent with the function of both HDAC3 and PROX1 as transcriptional repressors (Fig. 4d and Supplementary Data 3). Pathway analysis (Reactome) revealed the group of transcripts co-upregulated by HDAC3 and PROX1 loss to be highly enriched for regulation of lipid metabolism, including a number of important mediators of lipid synthesis and lipolysis (Fig. 4d). Correlation analysis comparing the frequency of HNF4α-dependent HDAC3–PROX1 binding sites at unchanged and HDAC3–PROX1 coregulated genes revealed a statistically significant relationship (chi-square with Yates correction, *P* < 0.0001). This association was consistent at lipid-related genes (Fig. 4e), and point to a strong correlation between co-upregulated lipid-related gene expression and the co-occupancy of HNF4α-dependent, HDAC3–PROX1 co-bound sites.

Transcriptional coregulation of a number of genes whose expression are critical to maintenance of lipid homeostasis, such as *G0s2* ^39^, *Elovl6* ^40^, *Mfsd2a* ^41^, and *Cidec* ^42^ were confirmed by qPCR following *Hdac3* knockout or *Prox1* knockdown (Supplementary Fig. 7). Upon examination, a number of these loci exhibited a strong overlap of HDAC3, PROX1, and HNF4α ChIP-seq signals, consistent with our genome-wide analysis (Fig. 4f and Supplementary Fig. 8a–d). In addition, we observed upstream co-bound regions that exhibited high H3K27 acetylation, suggesting that these sites may be putative enhancers. Indeed, upon loss of HDAC3 or PROX1, we observed an increase in HNF4α-dependent H3K27 acetylation at these putative enhancer regions. Moreover, analysis of nascent transcription, utilizing an existing global run-on sequencing (GRO-seq) data set in liver^43^, revealed bi-directional enhancer RNAs (eRNAs) at a number of co-bound regions. To determine whether these binding sites act as enhancers, we cloned the *G0s2* −17 kb and −1.7 kb binding sites into the pGL4 Luciferase reporter. Co-transfection of the enhancer reporter plasmid with an HNF4α expression vector revealed strong transcriptional induction by HNF4α at both the −17 kb and −1.7 kb sites, whereas mutation of the HNF4α binding site severely disrupted activation by HNF4α (Fig. 4g and Supplementary Fig. 9). Interestingly, expression of PROX1 significantly repressed HNF4α-mediated transcriptional activation, whereas it was unable to repress trans-activation induced by C/EBPα expression. These data indicate that the −17 kb, and to a lesser extent the −1.7 kb, binding sites act as HNF4α-controlled enhancers for the expression of *G0s2* and that HDAC3 and PROX1 act as direct repressors of these enhancers. Overall, our results indicate that the interaction between HDAC3 and PROX1 is critical for regulating liver metabolic gene expression and maintenance of hepatic lipid homeostasis.

## Discussion

In this study, we determined in vivo protein interactors of the class I histone deacetylase HDAC3 in mouse liver. This interactome revealed a number of binding partners in addition to confirming the well-established role of HDAC3 as a component of the NCoR complex^16, 17, 20^. Among these binding partners, we explored the functional interaction between HDAC3 and the Prospero-related homeobox protein PROX1. We determined that HDAC3 and PROX1 co-occupy a high number of genomic binding sites and that these binding sites correlate strongly with both the DNA-binding motif and the cistrome of HNF4α. Moreover, upon loss of HNF4α, a significant portion of HDAC3 and PROX1 co-occupied sites exhibit a profound loss of genomic binding. Depletion of PROX1 specifically in mature adult liver resulted in a marked increase in liver triglyceride content similar to that observed upon loss of HDAC3^12^, likely due to the dysregulation of a coregulated gene expression program important for lipid synthesis and lipolysis. Furthermore, we defined a liver enhancer responsible for controlling the expression of the lipolysis inhibitor G0S2, and showed that its transcriptional activation by HNF4α can be specifically repressed by PROX1. Overall, these results strongly suggest HDAC3 and PROX1 function in liver to corepress gene transcription important for maintenance of lipid homeostasis.

Protein–protein interactions are critical for determining biological functionality, including the control of chromatin function and gene expression, and can be strongly influenced when a binding partner is restricted to a specific cell type or tissue. Previous studies defining interacting partners for HDAC3 by mass spectrometry have not provided significant depth or elucidated tissue-specific factors bound with HDAC3 in vivo^15, 18, 44, 45^. Our strategy for identifying HDAC3 interactors in liver differs from methods that have been successful in cultured cells^46^ in two important ways. First, through co-expression of fusion-tagged HDAC3 and Cre recombinase in *Hdac3* ^*fl*/*f*^ animals via adeno-associated viral vectors (AAV8) under the control of the thyroxine binding globulin promoter (TBG), we were able to express our bait protein in vivo in hepatocytes lacking endogenous HDAC3. Second, we found that cross-linking isolated nuclei rather than whole liver depleted significant cytoplasmic and mitochondrial liver contaminants leading to reduced sample complexity and increased depth and sensitivity of our interacting protein mass spectrometric identifications. Nuclear isolation prior to functional assays, co-IP, and ChIP has been widely utilized in the transcriptional field^47^, most recently being employed for techniques such as GRO-seq^48^ and INTACT^49^. In addition, we have compared the cistromes of HDAC3 from standard ChIP-seq and those produced with our protocol and found them to be highly correlated (Pearson correlation, *R* = 0.781), suggesting that the nuclear isolation step is not having a major effect on the biological landscape that we are attempting to elucidate.

Similar to cross-linking methods developed for cultured cells^46^, the present method is improved in its ability to capture more transient interactions, including those at the genome. Of note, although previous work has shown the circadian nuclear receptor Rev-erbα and other nuclear receptors to be interacting partners for the HDAC3-containing NCoR complex^6, 21^, these proteins were not found in a previous HDAC3 proteomic screen performed in CEM T cells^45^. By contrast, the current study revealed several important HDAC3-bound nuclear receptors in liver including HNF4α, RXRα, Rev-erbα, and FXR, implicating HDAC3 in their functions. Our screen also revealed several non-nuclear receptor sequence-specific transcription factors such as C/EBPα, ChREBP, CREB1, and ETV6, possibly pointing to a broader role of HDAC3/NCoR in corepressing non-nuclear receptor transcription factors in liver. Alternatively, it is conceivable that our cross-linking-based sample preparation is capable of capturing targets of HDAC3 enzymatic activity. Indeed, a number of the interactors elucidated in our screen have been shown to be regulated by lysine acetylation including C/EBPα^50^, ChREBP^51^, CREB1^52^, and others, and it is therefore interesting to speculate that HDAC3 may regulate their functions through this mechanism.

Interaction of HDAC3 was also observed with several important nuclear complexes such as Integrator^53, 54^, Cohesin^55^, SWI/SNF^56^, and NuRD^57^, which have been implicated in the regulation of transcription through binding and functional control of gene-specific enhancers. Previous studies suggested that HDAC3 genomic binding correlates primarily with regions of active transcription^58^, which our results strongly support and extend to suggest that these components of the transcriptional machinery may be involved. It will be interesting to determine whether HDAC3 has a role as a bona fide member of these complexes or rather regulates their function through enzymatic or non-enzymatic mechanisms. The processes by which transcriptional activators and repressors act together at these enhancers to specifically tune gene expression remains an important unanswered question.

Although PROX1 has been shown to be critical for the development and specification of several tissues^25–29, 59^, its functions in the physiology of adult liver have not been determined. Our study provides evidence that PROX1 in adult liver helps maintain proper lipid homeostasis, as its depletion results in metabolic gene transcriptional changes and a strong upregulation of hepatic triglycerides. A previous study suggested that haploinsufficiency of *Prox1* in mice results in obesity^60^, and these animals exhibited an increase in liver lipids, which was suggested to be secondary to obesity. Our results suggest that the haploinsuffficiency of *Prox1* in hepatocytes might also contribute to the hepatosteatotic phenotype.

Unlike PROX1, which displays significant tissue specificity^31^, HDAC3 expression is relatively ubiquitous. Even so, PROX1 and HDAC3 are implicated in the control of several overlapping tissues such as pancreas, heart, central nervous system, and liver. Thus it is possible that, in these biological contexts, PROX1 provides tissue-specific functionality to HDAC3 through protein–protein interaction. HDAC3 has been implicated in beta cell function and the regulation of glucose homeostasis^61^. Interestingly, genome-wide association studies have revealed a CC variant SNP of *Prox1* (rs340841) that contributes to the control of fasting glucose levels and the development of diabetes^62, 63^. Perhaps in pancreatic beta cells, as in liver, HDAC3 and PROX1 form a complex to regulate gene expression involved in the control of organismal metabolism.

NAFLD is a disorder whose prevalence is increasing alongside metabolic syndrome, obesity, and type 2 diabetes and affects as high as 30% of the adult population in developed countries^1, 2^. As a major risk factor for liver inflammation (NASH) and scarring/fibrosis^3^, understanding the molecular mechanisms of liver function is of critical importance to development of therapies for this unmet medical need. Here, we have defined an interaction between HDAC3 and PROX1 that is nucleated at the genome by HNF4α, and elucidated the role of this repressor module in controlling hepatic triglyceride content by modulating lipid synthesis and lipolytic gene expression. These results highlight the importance tissue-specific corepressor interactions in maintaining liver metabolism and illustrate new pathways for therapeutic intervention.

## Methods

### Animal studies

Mice were housed in a temperature-controlled specific-pathogen-free facility under 12 h light/dark cycles (lights on at 0700 hours, off at 1900 hours). Adult male mice between the ages of 10–16 weeks old were used in all experiments unless otherwise indicated. AAV8 TBG Cre was intravenously injected at 1.5 × 10^11^ GC per mouse to induce hepatocyte-specific gene knockout, using AAV8 TBG GFP as a negative control. For MS experiments AAV8 TBG HDAC3-HA or control AAV8 TBG HA-EGFP vectors were injected at 5 × 10^10^ GC per mouse in combination with either AAV8 TBG Cre or AAV TBG GFP, respectively. All mice in an experiment received equal total dosages of AAV vectors by supplementing with AAV8 TBG empty or AAV8 TBG GFP vectors where appropriate. All mice were killed by CO_2_ inhalation followed by tissue harvest at 2 weeks after viral injection at ZT10 except indicated otherwise. All animal procedures followed the guidelines of the Institutional Animal Care and Use Committee of the University of Pennsylvania.

### Constructs and viral vectors

AAV8 TBG viral constructs for the expression of HA-EGFP and HDAC3-HA were generated by PCR cloning and Gibson Assembly. All constructs were sequence verified by Sanger sequencing at the Penn Genomic Analysis Core. Adeno-associated viruses were produced and purified by the University of Pennsylvania Vector Core. AAV-based knockdown vectors were generated through Gibson cloning of *Egfp* and the UltramiR mir-30 scaffold^64^ into a modified AAV8 TBG vector containing a downstream Woodchuck Hepatitis Virus Posttranscriptional Regulatory Element (WPRE). Two target sequences used for *Prox1* knockdown, determined using the shERWOOD algorithm^64^, were: (TTCAGAGCAGGATGTTGAATA) and (GAGAAGGCAGCAACAAAGAAA). The control shRNA sequence targeting Luciferase was (CGCTGAGTACTTCGAAATGTC). For in vivo knockdown experiments mice received 1 × 10^12^ GC per mouse of each *Prox1* targeting virus or 2 × 10^12^ GC per mouse of the control shLuciferase virus. For enhancer luciferase reporter vectors, enhancers for *G0s2* were selected by comparing H3K27Ac and GRO-seq tracks (GEO Accession number GSM1437738)^43^ with binding sites for HDAC3, PROX1, and HNF4α. Enhancer regions of 200 bp were cloned into the MCS of the pGL4 luciferase reporter system (Promega). pRL-SV40 renilla was used as a control for firefly luciferase expression. Primers used in this study can be found in Supplementary Data 4.

### Liver triglyceride measurement and oil red O staining

For measuring triglyceride, livers were lysed in lysis buffer (140 mM NaCl, 50 mM Tris, and 1% Triton X-100, pH 8) followed by triglyceride assay using LiquiColor kit (Stanbio). Oil red O staining was performed by the University of Pennsylvania Center for Molecular Studies in Digestive and Liver Disease Molecular Pathology & Imaging Core.

### Cell culture and luciferase assay

HEK 293T cells purchased from ATCC were maintained in DMEM medium containing 10% FBS and 100 µg ml^−1^ Penicillin/streptomycin at 5% CO_2_ and 37 °C. Transfections of plasmids for the expression of proteins and luciferase reporter constructs were performed using Fugene 6 (Roche). For luciferase assays, after 18–24 h of transfection, cells were washed once with PBS followed by lysis in passive lysis buffer (Promega). Lysates were analyzed for firefly and control renilla luciferase activity using the Dual-Luciferase Reporter Assay System (Promega) according to the manufacturer’s instructions and read on a microplate reader equipped with a dual-injection system (BioTek).

### Immunoprecipitation and western blotting

For western blot analysis of total lysates, samples were lysed in a TissueLyser (Qiagen) in radioimmunoprecipitation assay buffer (RIPA buffer) supplemented with complete EDTA-free protease inhibitor (Roche) and 1 mM PMSF. Samples were resolved by Tris-glycine SDS-PAGE (Biorad), transferred to nitrocellulose membrane (Biorad), and blotted with the indicated antibodies. Antibodies for western blotting were anti-PROX1 (Millipore, 07-537, 1:1000), anti-HDAC3 (GeneTex, GTX113303, 1:1000), anti-HNF4α (Santa Cruz, sc-8987, 1:500), anti-HA 3F10 High Affinity (Roche, 12013819001, 1:5000), anti-FLAG M2 HRP (Sigma, A8592, 1:10,000), anti-Actin HRP (Santa Cruz, sc-1616 HRP, 1:5000), anti-HSP90 (Cell Signaling, 4874, 1:1000), and anti-rabbit HRP (Cell Signaling, 7074, 1:10,000). For immunoprecipitation followed by western blot livers were cross-linked with 2 mM dithiobis(succinimidyl propionate), DSP (Pierce) in PBS at room temperature for 30 min, quenched with glycine, washed with ice cold PBS, and subsequently lysed in RIPA buffer containing protease inhibitors and PMSF. Samples were pre-cleared with protein A sepharose CL-4B (GE Healthcare), and incubated with either anti-HA agarose (Sigma, A2095) or with anti-HDAC3 (GeneTex, GTX113303, 10 μg) or anti-PROX1 (Millipore, 07-537, 10 μg) antibodies and captured with TrueBlot anti-Rabbit IgG agarose (Rockland). Immunoprecipitates were washed five times with RIPA and eluted with SDS loading dye. For in vitro coimmunoprecipitation analysis, HEK 293T cells were transfected with pcDNA FLAG-NCOR1 or PROX1 vectors with Fugene 6 (Promega). For domain mapping of PROX1 interaction with HDAC3, pcDNA FLAG-tagged PROX1 mutants, HDAC3-HA and EGFP were transfected as indicated. At 72 h after transfection, cells were washed with PBS and lysed in IP buffer (50 mM Tris 7.4, 150 mM NaCl, and 0.5% NP-40) containing protease inhibitors and 1 mM PMSF. Samples were pre-cleared with protein A sepharose CL-4B (GE Healthcare), and incubated with anti-FLAG M2 resin (Sigma, A2220). Immunoprecipitates were washed five times with IPLS and eluted with SDS loading dye for western blot analysis. Uncropped scans of western blots are shown in Supplementary Fig. 10.

### Mass spectrometry

Livers from *Hdac3* ^fl/fl^ animals transduced with AAV8 TBG HDAC3-HA and AAV8 TBG Cre or AAV8 TBG HA-EGFP and AAV8 TBG EGFP were harvested after 2 weeks of infection. A 250 mg sample of liver was dissected, dounced 10 strokes with pestle A in 15 ml swelling buffer (10 mM Tris-HCl pH 7.4, 2 mM MgCl_2_, 3 mM CaCl_2_) supplemented with 1 mM PMSF, and incubated for 20 min on ice. Swelled cells were dounced 15 strokes with pestle B, an additional 15 ml swelling buffer with PMSF was added, filtered through a 100 µm cell strainer, and spun at 400×*g* for 10 min to collect cells. Cells were resuspended in 5 ml swelling buffer with 10% glycerol and PMSF and an additional 5 ml of swelling buffer supplemented with 10% glycerol, 1% NP-40, and 1 mM PMSF was added slowly while vortexing. Following lysis, nuclei were washed 1 time with PBS and subsequently fixed with 1% formaldehyde diluted in PBS for 15 min at room temperature. Cross-linked samples were quenched with glycine, washed once with PBS, and resuspended in 1 ml NCB (20 mM Tris-HCl pH 8, 100 mM KCl, 5 mM MgCl_2_, 10% glycerol, 0.1% NP-40, 1 mM DTT) supplemented with complete EDTA-free protease inhibitor tablet (Roche) and 1 mM PMSF. Lysis was allowed to proceed for 1 h followed by sonication with a probe sonifier (Branson). Cross-linked chromatin extracts were cleared by centrifugation and where indicated samples were treated with 1000 Kunitz units of either micrococcal nuclease (NEB) or Benzonase nuclease (Sigma) for 30 min at 30 °C. Extracts were immunoprecipitated with anti-HA agarose (Sigma, A2095) overnight, washed three times in NCB supplemented with protease inhibitors and 1 mM PMSF, washed two times in NCB, washed two times in HPLC-grade water, eluted with 10% ammonium hydroxide diluted in HPLC-grade water, and dried to completion in a SpeedVac (Eppendorf).

Samples were prepared for MS^65^. Briefly, after reduction/alkylation, samples were digested with LysC (Wako) for 2 h followed by Trypsin (Promega) at 37 °C overnight. Samples were resuspended in 1% acetic acid, and desalted with C_18_ stage tips (Empore, 2215). EASY-nanoLC (Thermo Fisher Scientific) was configured with a 75 µm ID × 17 cm Reprosil-Pur C_18_-AQ (3 µm; Dr Maisch GmbH, Germany) nano-column and coupled with an Orbitrap Fusion mass spectrometer (Thermo Fisher Scientific). Full scan MS spectrum (*m*/*z* 360−1600) was performed in the Orbitrap with a resolution of 120,000 (at 200 *m*/*z*). Fragmentation was performed with higher energy collisional dissociation (HCD) and a maximum injection time of 120 ms. MS/MS data were collected in centroid mode in the ion trap mass analyzer. Peptides were identified using MaxQuant (v1.5.3.30) using the Mus Musculus UniProt FASTA database (March 2016, Q921N8 added) and MaxQuant contaminants FASTA with an FDR < 1% at the peptide spectrum match and protein levels. Protein abundance was deconvoluted from peptide intensity using the intensity-based absolute quantification (iBAQ)^24^ algorithm, followed by log_2_ transformation, normalization by the median intensity, and missing values imputed (width 0.25, downshift 2.0) using Perseus (v1.5.5.3) (Supplementary Data 1). Significance was estimated using a two-tailed homoscedastic *t*-test (*P*-value < 0.05).

### RT-qPCR and RNA-seq

Total RNA was extracted using TRIzol (Invitrogen) and RNeasy mini kit (Qiagen). Contaminating DNA was removed using the RNase-Free DNase Set (Qiagen) on-column digestion protocol per manufacturer’s instructions. RT-qPCR was performed with the High Capacity RT kit (Thermo Fisher Scientific), Power SYBR Green PCR Master Mix (Thermo Fisher Scientific), and a QuantStudio™ 6 Flex Real-Time PCR System (Thermo Fisher Scientific) using absolute quantification method with standard curves. *36b4* (*Arbp*) was used as housekeeping control. RNA-seq libraries were generated using the Tru-seq kit (Illumina). Raw reads were aligned to the mm9 reference genome using Tophat version 2.1.9 using the parameters recommended by the original author. Differential expression analysis was performed using CuffDiff 2 using default parameters.

### ChIP-qPCR, ChIP–reChiP, and ChIP-seq

For ChIP and ChIP-seq^6, 66^, livers were mildly dissociated by dounce with pestle A for six strokes in PBS containing 1% formaldehyde and rocked for 15 min, quenched with glycine, washed with PBS, and sonicated with a probe-type sonifier (Branson) in RIPA supplemented with protease inhibitors and PMSF. Sonicated extracts were immunoprecipitated with antibodies for HDAC3 (Abcam, ab7030, 10 μg), PROX1 (Millipore, 07-537, 10 μg), HNF4α (Santa Cruz, sc-8987, 10 μg), or H3K27Ac (Abcam, ab4729, 2 μg), and captured with bovine serum albumin blocked CL-4B protein-A sepharose beads (GE). PROX1 ChIP-seq was validated by comparing results of ChIP-seq with two different PROX1 antibodies raised against separate epitopes (Millipore, 07-537 and Proteintech, 51043-1-AP), which showed a highly significant Pearson correlation (*R* = 0.96). The HNF4α and HDAC3 antibodies used for ChIP in this study have been validated in the literature^67, 68^.

ChIP–reChIP from three biological replicate livers was performed essentially as standard ChIP, except following the first IP chromatin-protein complexes were eluted in 1% SDS with 10 mM DTT for 15 min at 65 °C. Subsequent to elution of the first ChIP, complexes were re-diluted in 10 volumes RIPA supplemented with protease inhibitors (Roche), PMSF (Sigma), 5 mg ml^−1^ BSA, and 2 μg Lambda DNA/HindIII (NEB), followed by a second ChIP with the either anti-rabbit IgG (Cell Signaling Technologies, #2729) or the indicated antibody.

For ChIP-seq, ChIP was performed independently on livers from different mice (*n* = 3). The precipitated DNA samples were barcoded and amplified according Illumina guide protocols, followed by deep sequencing on a HiSeq 2000 (Illumina). Sequencing reads of biological replicates for PROX1 and HDAC3 were aligned to the mm9 genome using Bowtie v0.12.7. Duplicate reads were removed and replicates were pooled using HOMER v4.7. Genome browser tracks were generated and peaks were called using HOMER with default parameters and IgG as input. Peaks more than threefold over input and >2 rpm in PROX1 and >1.5 rpm in HDAC3 were used for further downstream analyses. Venn diagram was generated using bedtools v2.26.0 and Vennerable R package with peaks overlapping at least 50% and having at least 1 rpm in the other ChIP-seq. Motif analyses were performed with HOMER using 200 bp peak windows. Gene ontology was performed using Reactome 2016. Track visualization was performed using the Integrated Genomics Viewer (Broad Institute).

Sequencing reads of biological replicates for PROX1, HDAC3, and HNF4α in *Hnf4α* ^fl/fl^ and *Hnf4α* conditional liver knockout animals were processed similarly. Previously published C/EBPα ChIP-seq (GEO Accession numbers GSM1816821 and GSM1816822, replicates combined)^69^ and C/EBPβ ChIP-seq (GEO Accession number GSM1446070)^66^ were re-processed using the same parameters. Peaks >1 rpm for PROX1 and HDAC3 and >2 rpm for HNF4α were used for further downstream analyses. Scatter plots and box plots were generated using the HOMER annotatePeaks command and R.

### Statistical methods

Data are presented as mean ± s.d. unless otherwise stated. Microsoft Excel software, GraphPad Prism 7, MathWorks MATLAB, or R was utilized for all graphing and statistical tests. For comparison between two groups, two-tailed unpaired Student’s *t*-test were utilized unless otherwise stated, where *P* < 0.05 was considered statistically significant and the significance is marked by *P < 0.05, **P < 0.01, and ***P < 0.001 unless otherwise noted. Wilcoxon–Mann–Whitney test was performed for statistical comparison between HNF4α binding and C/EBPα or C/EBPβ binding in the HDAC3–PROX1 down peaks in *Hnf4α* liver KO vs.HDAC3–PROX1 unchanged peaks in *Hnf4α* liver KO (Supplementary Fig. 5c) and exact *P*-values were reported. For correlation analysis of HDAC3–PROX1 coregulated genes with HNF4α-dependent binding sites, a χ^2^ contingency table with Yates correction was used. All other statistical comparisons for two groups of peaks were done using Pearson’s χ^2^ contingency table tests and *P*-values were reported for each pair. All statistical tests are fully described in figure legends. The required sample size was calculated based on similar experiments and analyses carried out previously. The number of animals in each experiment is stated in the respective figure legends.

### Data availability

The data discussed in this publication have been deposited in NCBI’s Gene Expression Omnibus^70^ and are accessible through GEO Series accession number GSE90533 (https://www.ncbi.nlm.nih.gov/geo/query/acc.cgi?acc=GSE90533). All mass spectrometry data reported here have been deposited in Chorus under ID number 1251. All other data that support the findings of this study are available from the corresponding author upon reasonable request.

## Electronic supplementary material

Supplementary Information

Supplementary Data 1

Supplementary Data 2

Supplementary Data 3

Supplementary Data 4
