# Supplementary material for: An HDAC3-PROX1 corepressor module acts on HNF4α to control hepatic triglycerides
Source: Nat Commun. 2017 Sep 15;8:549. doi: 10.1038/s41467-017-00772-5 (PMC5601916; doi:10.1038/s41467-017-00772-5)

Description of Supplementary Files

File Name: Supplementary Information
Description: Supplementary Figures

File Name: Supplementary Data 1
Description: (Related to Fig. 1b). nLC-MS/MS analyses (MaxQuant) of all study associated experiments.

File Name: Supplementary Data 2
Description: (Related to Fig. 1c). nLC-MS/MS analyses of HDAC3 high-confidence interacting proteins.

File Name: Supplementary Data 3
Description: (Related to Fig. 4d). Relative transcript levels from RNA-seq of Hdac3 liver KO vs wild-type and shProx1 vs shLuciferase.

File Name: Supplementary Data 4
Description: PCR primers used in this study.

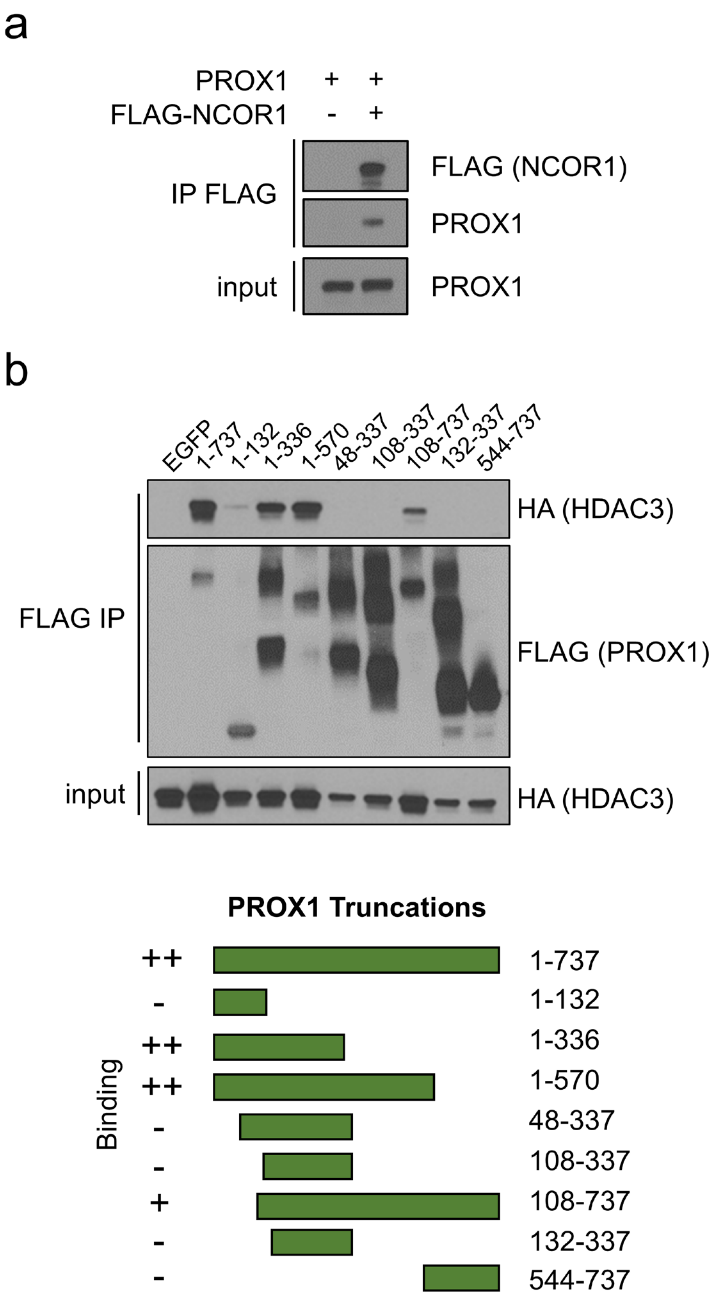

Supplementary Figure 1. Interrogation of PROX1 interaction with HDAC3-NCoR. (**a**) Co-immunoprecipitation western blot analysis of FLAG-NCOR1 co-expressed with PROX1 in HEK 293T cells. (**b**) Domain mapping to identify regions of PROX1 required for interaction with HDAC3. FLAG-tagged PROX1 truncations were co-expressed with HDAC3-HA in HEK 293T cells and subjected to FLAG immunoprecipitation followed by western blotting.

a

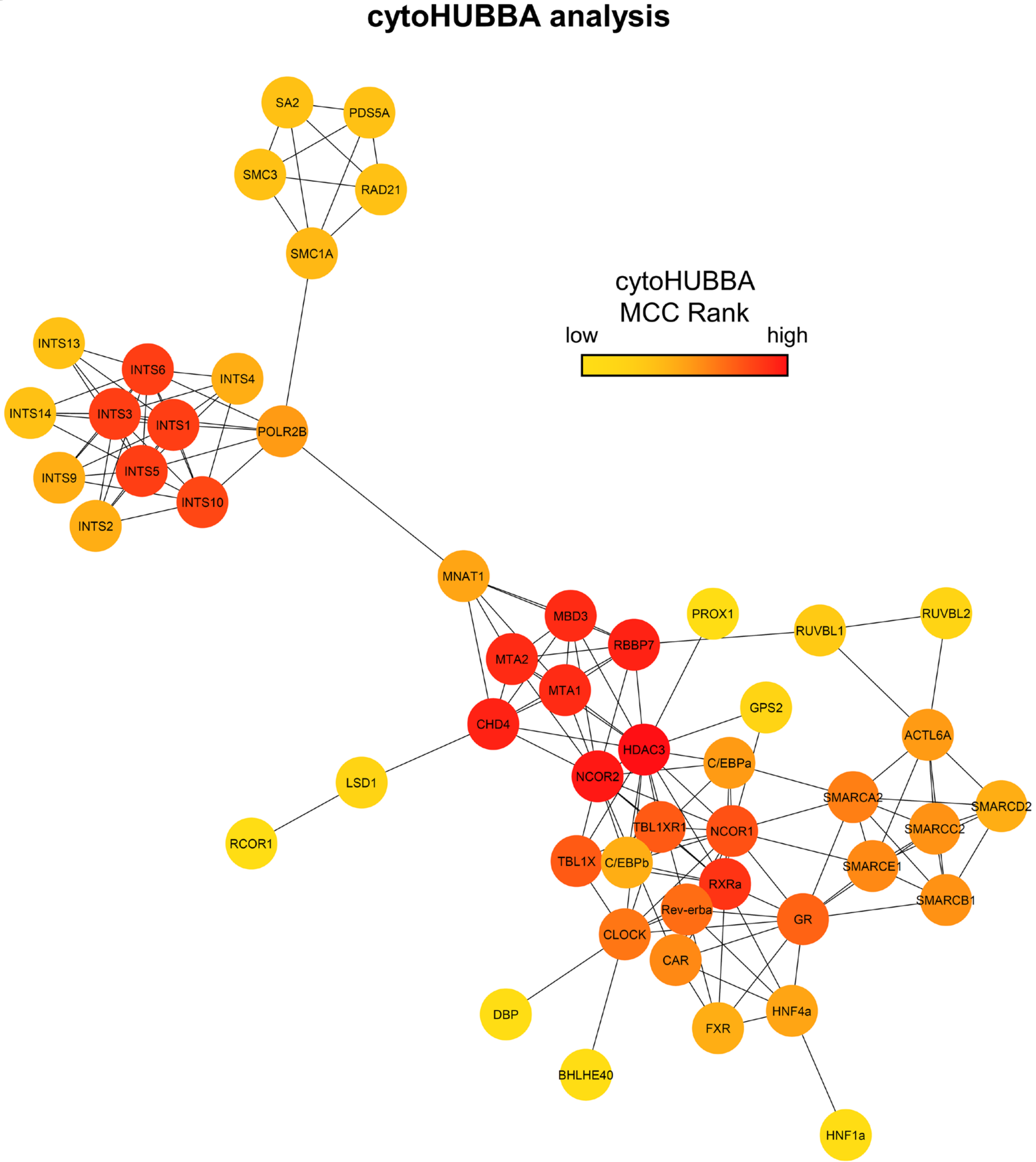

Supplementary Figure 2. HDAC3 interactome topology analysis. (**a**) cytoHUBBA analysis using the maximal clique centrality (MCC) of HDAC3 interactors annotated from STRING. Active interaction sources include experiments and databases with a minimum interaction score of 0.6. Nodes are colored by rank order.

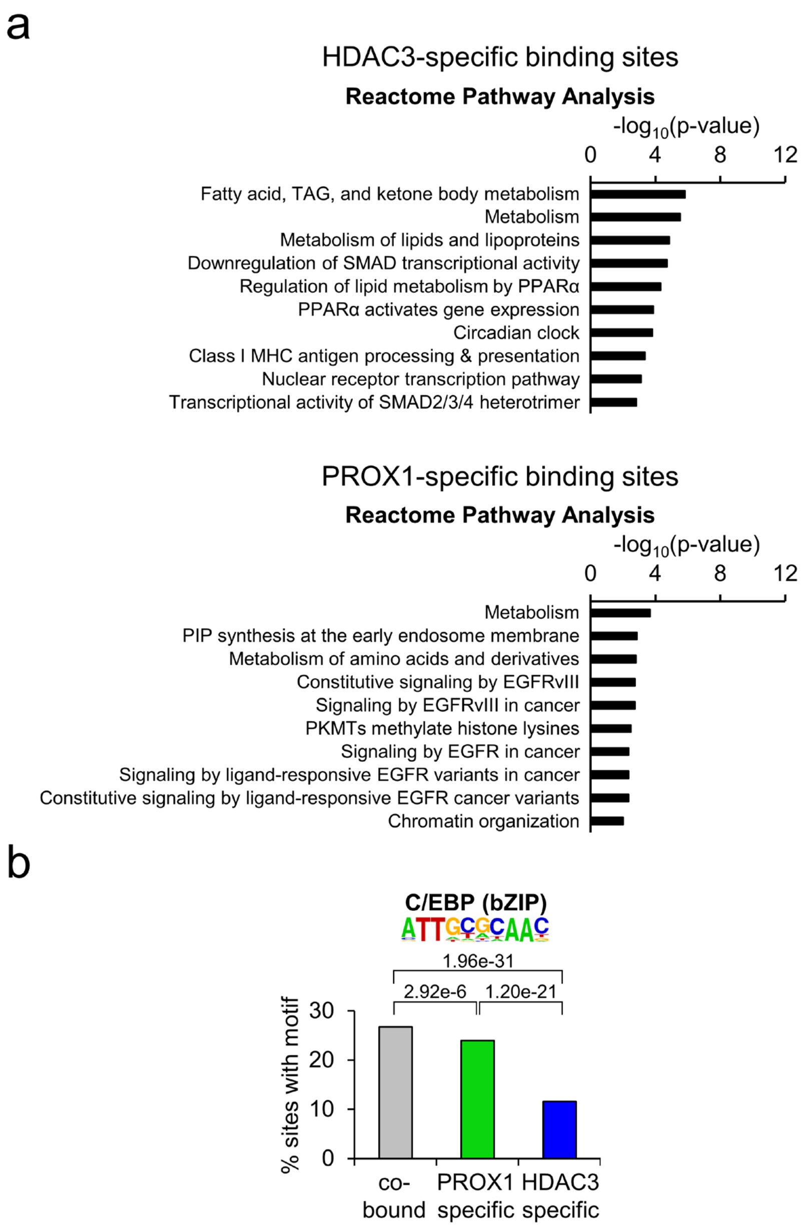

Supplementary Figure 3. HDAC3 and PROX1 supporting cistromic analysis. (**a**) Reactome analysis of the nearest genes within 100kb from the top 1000 HDAC3-specific and PROX1-specific peaks. (**b**) HOMER motif enrichment analysis of the C/EBP (bZIP) motif at overlapping and non-overlapping peaks determined in **Fig. 2a**. Numbers above brackets indicate p-values, Chi-squared test.

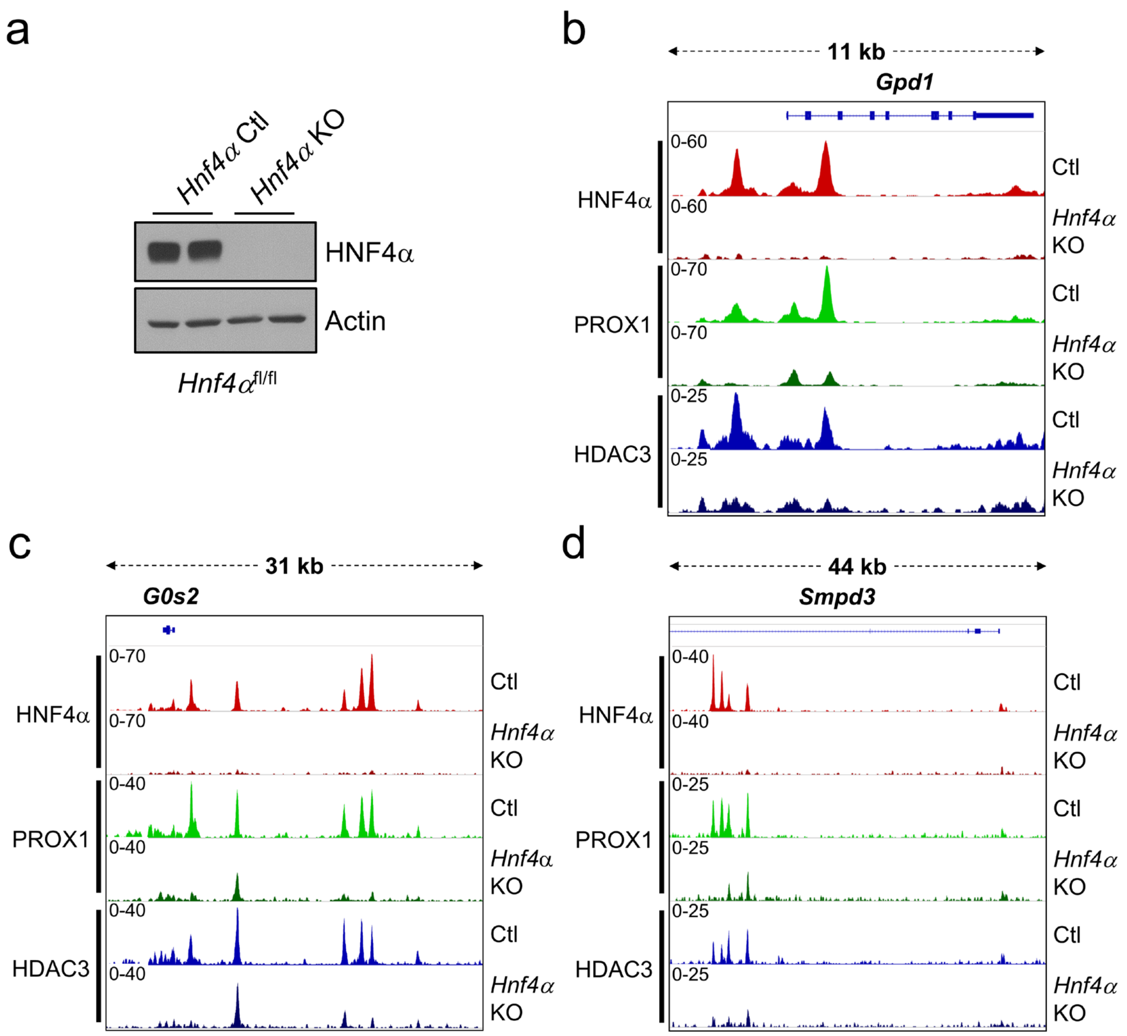

Supplementary Figure 4. HDAC3, PROX1, and HNF4α genomic binding in wild-type and *Hnf4α* knockout liver. (**a**) Western blot confirming HNF4α KO. (**b-d**) Representative browser tracks at lipid-related genes of HNF4α, PROX1, and HDAC3 ChIP-seq in *Hnf4α*$^{fl/fl}$ livers infected with AAV8 TBG *Egfp* (Ctl) or *Cre* (*Hnf4α* KO). Indicated scales are in RPTM.

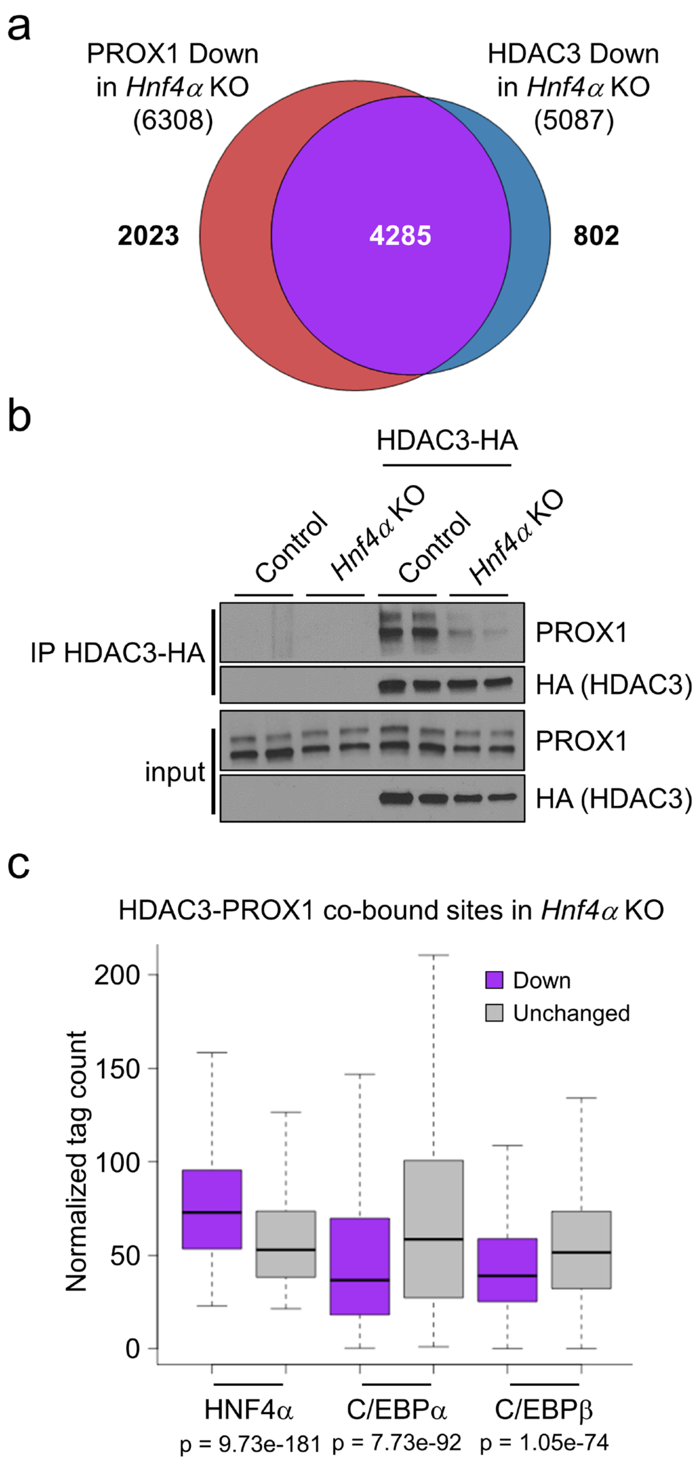

Supplementary Figure 5. HNF4α recruits the HDAC3-PROX1 corepressor module in liver. (**a**) Venn diagram displaying overlap (at least 50% and 1 rpm) of red and blue regions from **Fig. 3d**. (**b**) Co-immunoprecipitation western blot of HDAC3-HA in control and HNF4α KO liver. (**c**) Box and whisker plot indicating the ChIP-seq binding strength of HNF4α, C/EBPα, and C/EBPβ at HDAC3-PROX1 sites that are either down upon loss of HNF4α (purple) or unchanged (gray) (Wilcoxon-Mann-Whitney test).

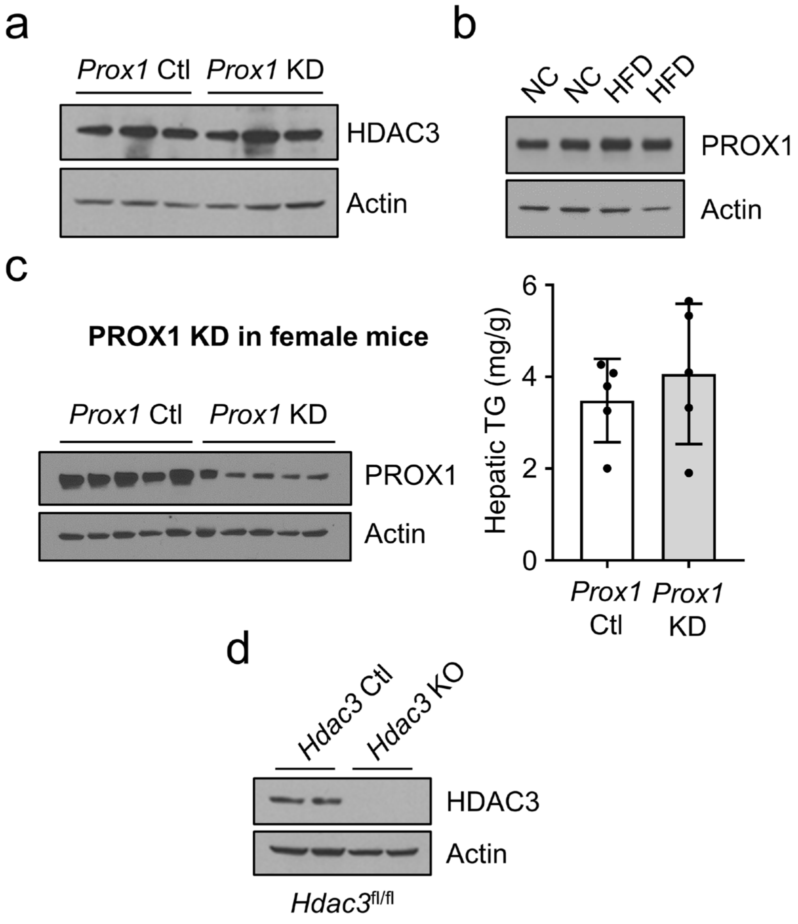

Supplementary Figure 6. Evaluation of PROX1 knockdown liver. (**a**) Western blot of HDAC3 in control and PROX1 KD liver. (**b**) Western blot showing PROX1 levels in male mice at 18 weeks of age fed for 12 weeks with normal chow diet (NC) or high fat diet (HFD). (**c**) Western blot and liver triglyceride levels of PROX1 KD in female mice (n=5) 6 weeks post injection. (**d**) Western blot validating HDAC3 KO.

a

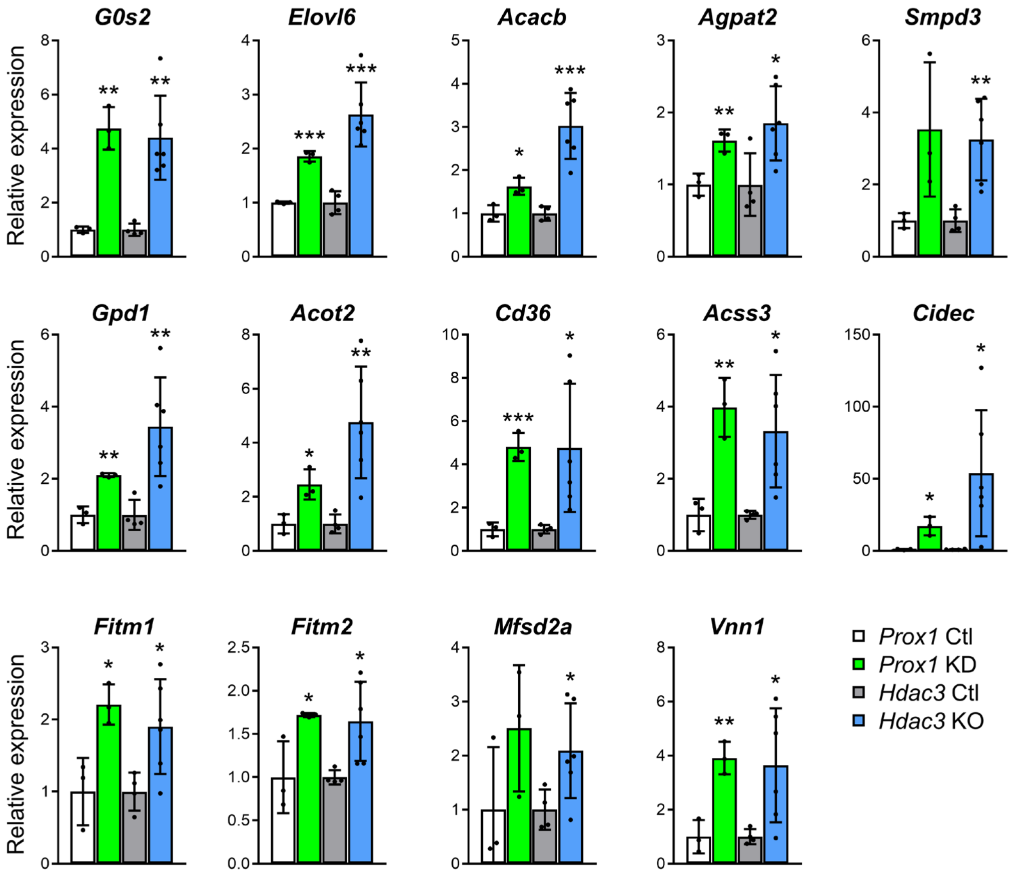

Supplementary Figure 7. Gene expression in HDAC3 and PROX1 ablated livers. (**a**) Confirmation of up-regulated lipid-related genes upon loss of HDAC3 (Ctl n=4, KO n=6) or PROX1 (Ctl n=3, KD n=3) by qPCR. Data are presented as mean±s.d. Two-tailed unpaired Student's t-test, *p<0.05, **p<0.01, ***p<0.001.

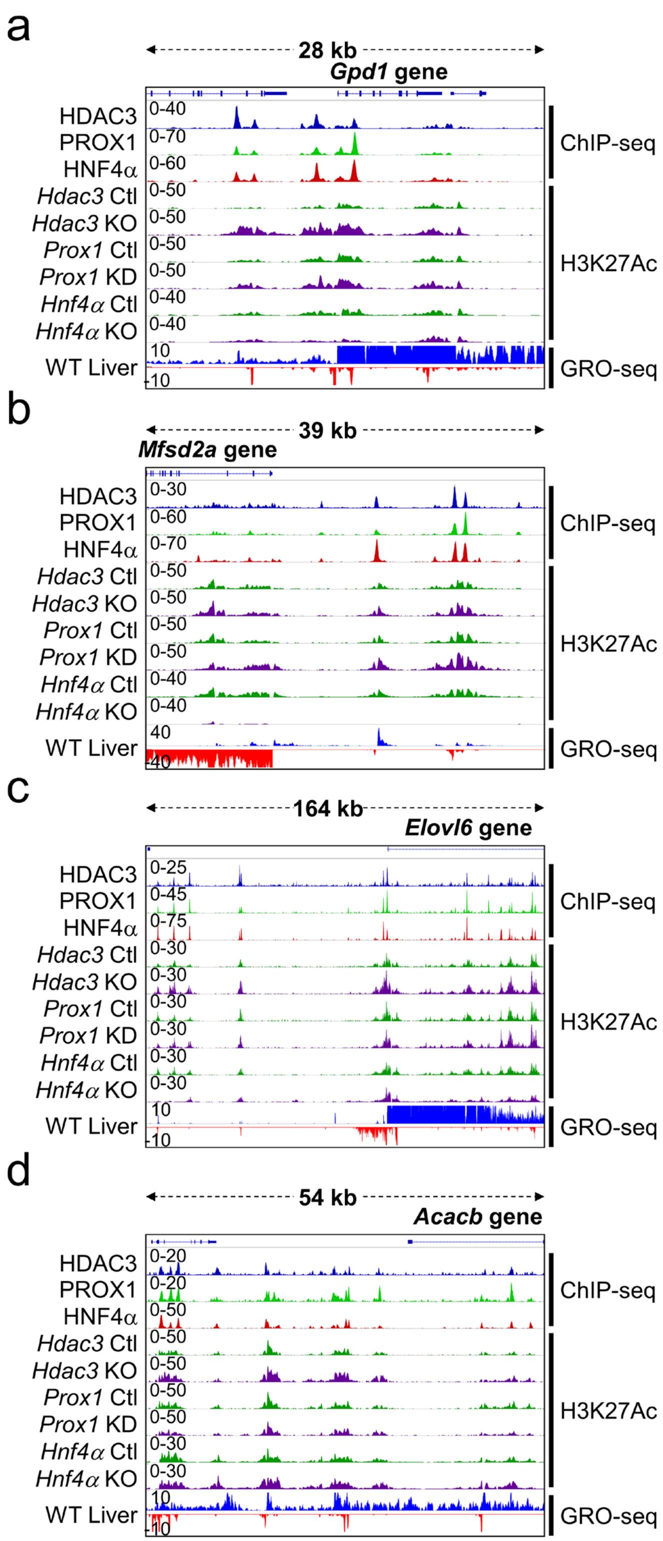

Supplementary Figure 8. Genomic binding and nascent transcription near lipid-related genes coregulated by HDAC3-PROX1 (**a-d**) Example ChIP-seq and GRO-seq browser tracks at lipid-related gene loci. Indicated scales are in RPTM.

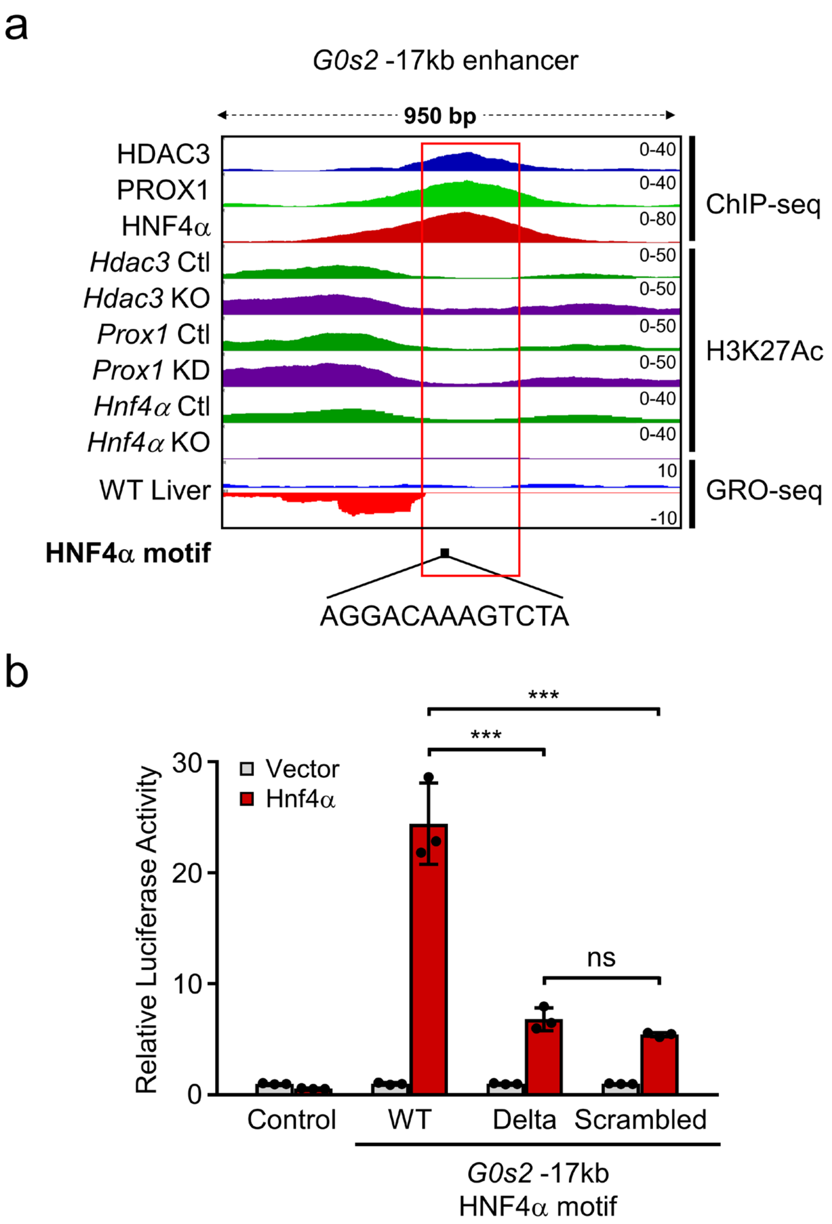

Supplementary Figure 9. HNF4α-induced enhancer function is dependent on an intact HNF4α DNA binding motif. (**a**) Track of *G0s2* locus with identified HNF4α motif. Indicated scales are in RPTM. (**b**) Luciferase assay (n=3) indicating transcriptional response to expression of HNF4α at wild-type and HNF4α DR1 mutant *G0s2* enhancer as indicated**.** Data are presented as mean±s.d, two-tailed unpaired Student's t-test, *p<0.05, **p<0.01, ***p<0.001, ns not significant.

Uncropped Figure 1d

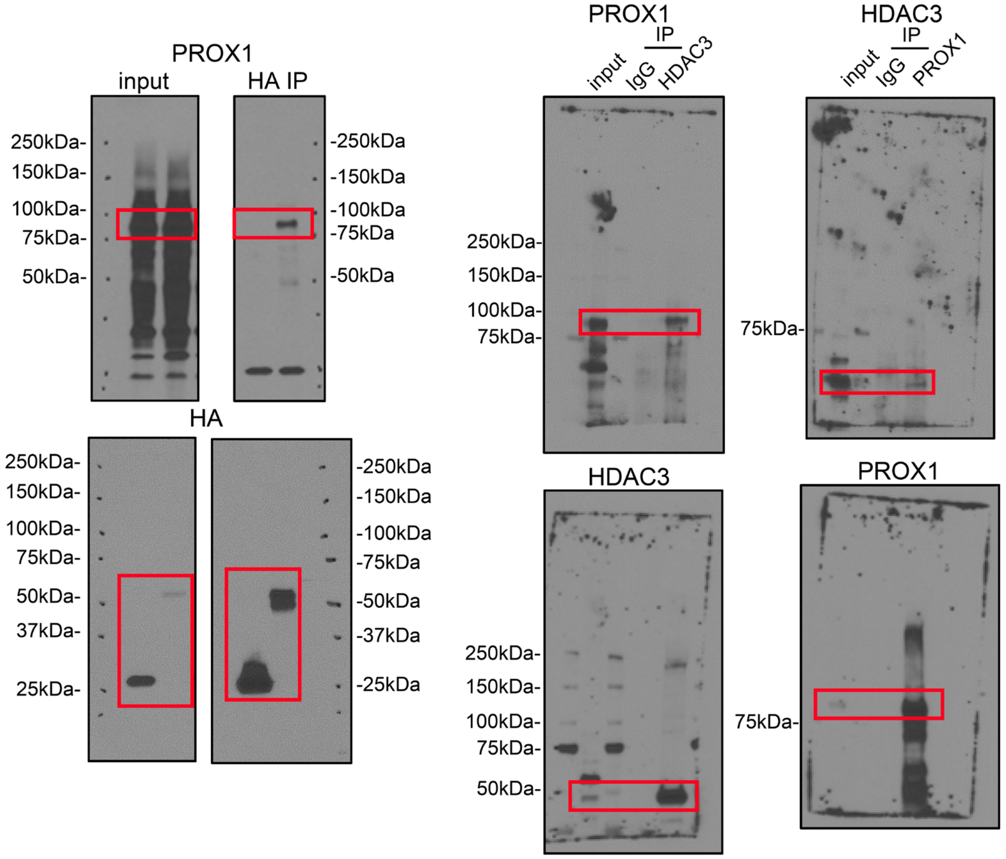

Uncropped Figure 4a

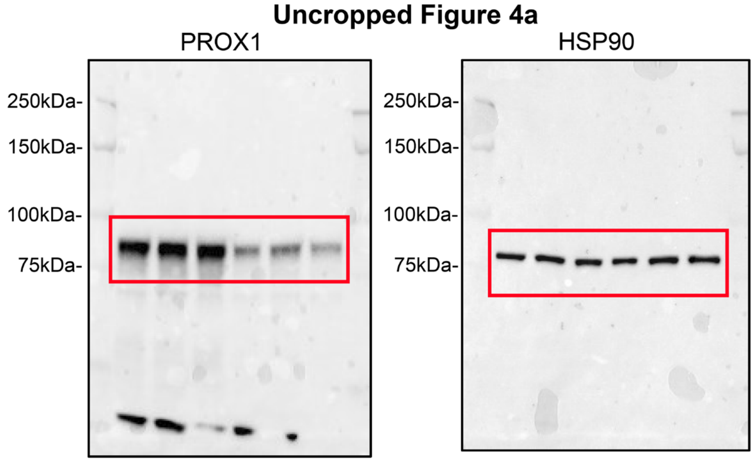

Supplementary Figure 10. Uncropped western blot scans. Uncropped western blot scans corresponding to indicated figures. Red box indicates region displayed in the corresponding figure.

Supplementary Figure 10 (continued).

Uncropped Supplementary Figure 1a

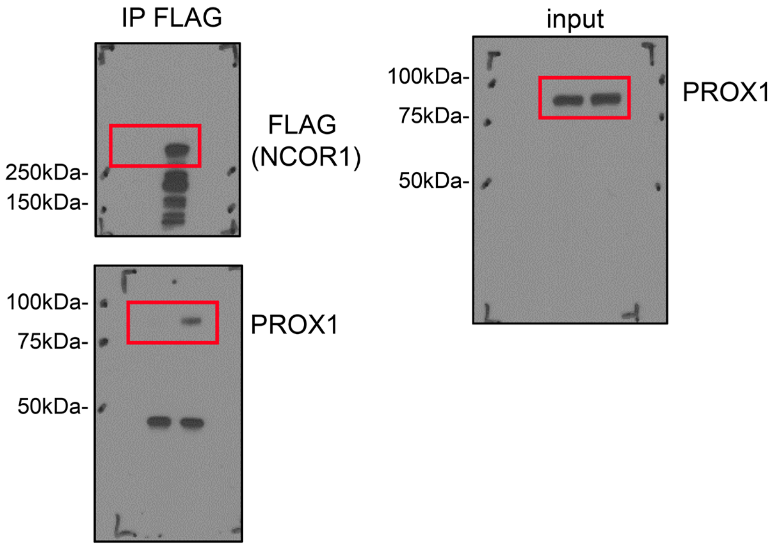

Uncropped Supplementary Figure 1b

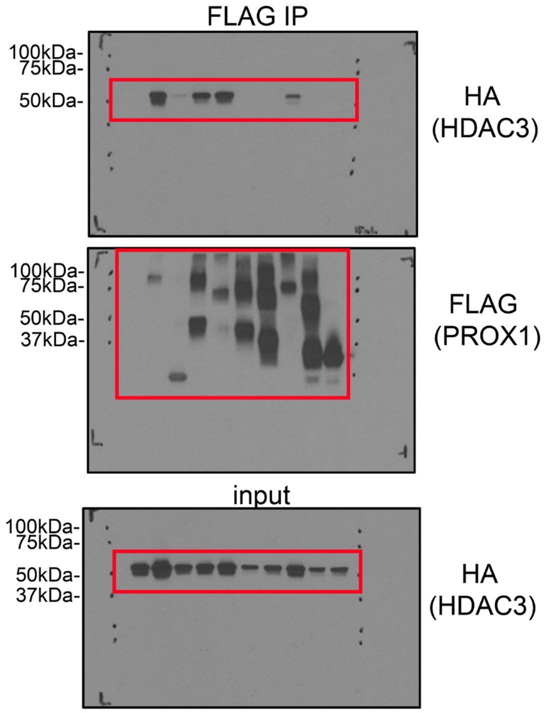

Uncropped Supplementary Figure 4a

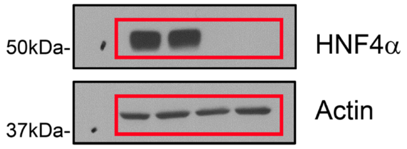

Supplementary Figure 10 (continued).

Uncropped Supplementary Figure 5b

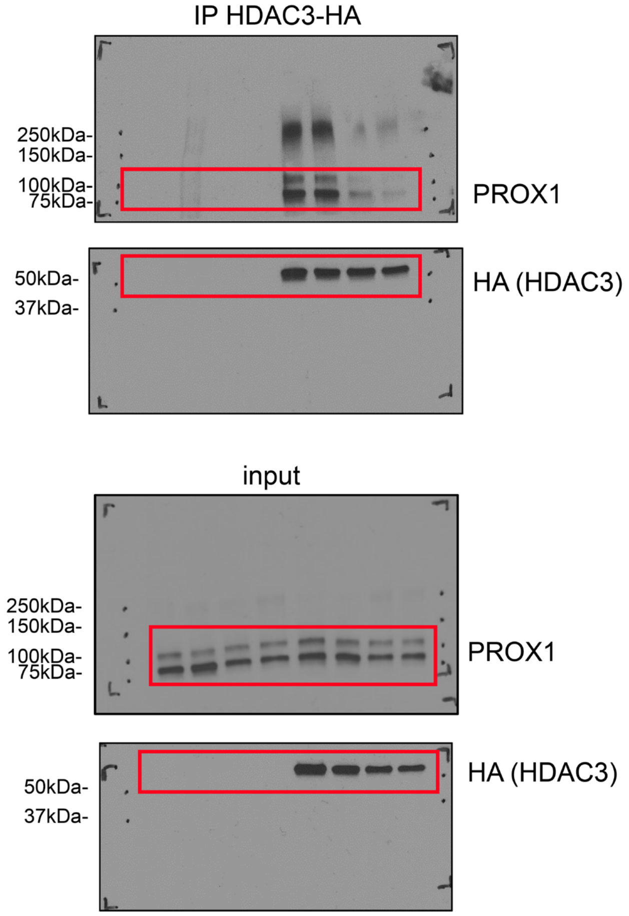

Supplementary Figure 10 (continued).

Uncropped Supplementary Figure 6a

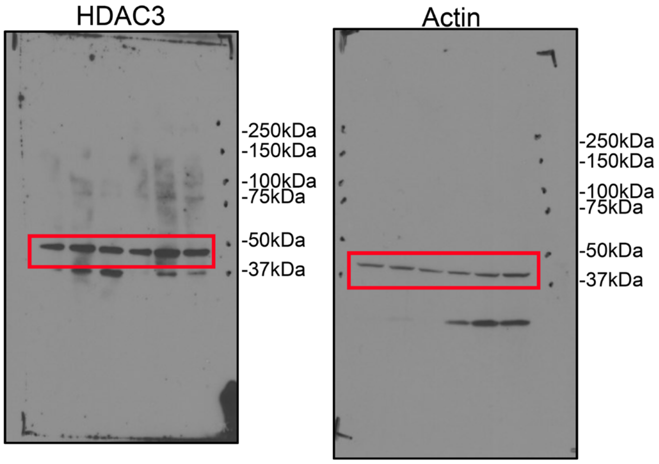

Uncropped Supplementary Figure 6b

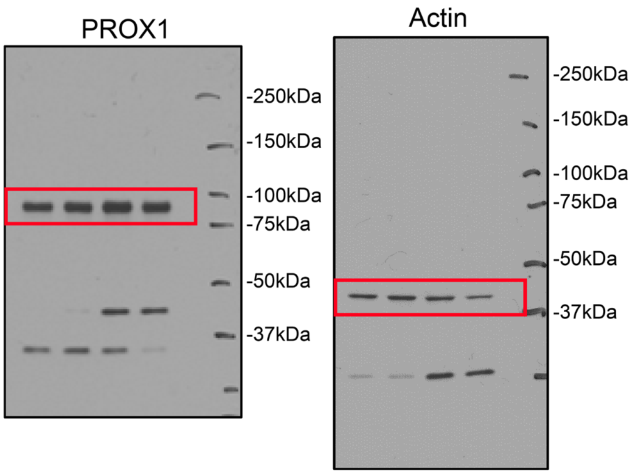

Supplementary Figure 10 (continued).

Uncropped Supplementary Figure 6c

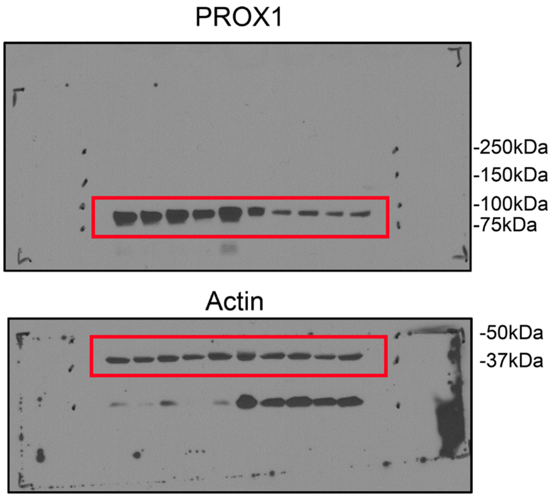

Uncropped Supplementary Figure 6d

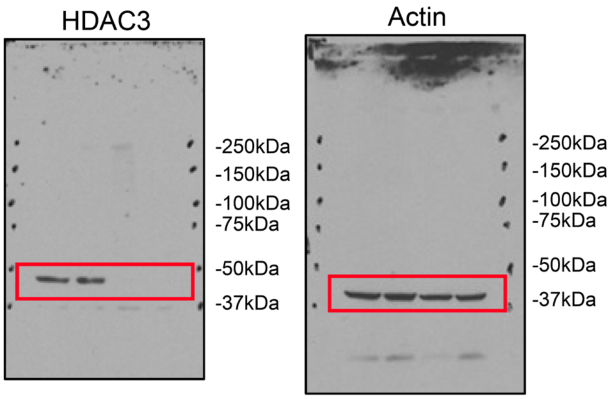

Supplement: Supplementary file 1 — Supplementary Information [file 41467_2017_772_MOESM1_ESM.pdf]
